# Metachronous primary lung adenocarcinomas harboring distinct *KRAS* mutations

**DOI:** 10.1111/1759-7714.13458

**Published:** 2020-05-16

**Authors:** Yan Hu, Siying Ren, Chen Chen, Qingchun Liang, Fenglei Yu, Wenliang Liu

**Affiliations:** ^1^ Department of Thoracic Surgery Second Xiangya Hospital of Central South University Changsha China; ^2^ Department of Respiratory and Critical Care Medicine Second Xiangya Hospital of Central South University Changsha China; ^3^ Department of Pathology Second Xiangya Hospital of Central South University Changsha China

**Keywords:** *KRAS* mutation, multiple metachronous primary lung cancers, next generation sequencing

## Abstract

To distinguish whether multiple lung nodules represent multiple primary lung cancers (MPLC) or intrapulmonary metastases (IPM) is crucial for staging and subsequent therapy. We herein present the first report of a patient with two simultaneously resected metachronous lung adenocarcinomas in the right upper lobe, each with a distinct driver mutation in the *KRAS* gene identified by targeted next generation sequencing (NGS). The nodules appeared chronologically metachronous, with a 3.7 year interval. Histopathology showed two histologically identical adenocarcinomas, without lymph node metastases. It was hard to decide whether they should be classified as either MPLC or IPM based only on the clinicopathological criteria. Sequencing further revealed distinct *KRAS* mutation in each tumor, with one tumor harboring the *KRAS*‐G12C mutation, and the other tumor harboring the *KRAS*‐Q61H mutation. Incorporation of the molecular data cleared the confusion with regard to staging and spared this patient from adjuvant therapy. This case highlights that molecular profiling allows for better differentiation between MPLC and IPM than histopathology alone.

**Key points:**

1. To the best of our knowledge, this is the first case of multiple primary lung cancers harboring distinct *KRAS* mutations.
2. The case highlights the importance of incorporating molecular profiling using NGS along with the clinicopathological criteria in classifying multiple lung tumors.

## Introduction

Multiple lung nodules are being diagnosed with an increased frequency because of the development of high‐quality computed tomography (CT) imaging over the past decade.^1^ The crucial issue regarding multiple lung nodules is whether they can be classified as either intrapulmonary metastases (IPM) or multiple primary lung cancers (MPLC), as it is important for both prognostication and determining adjuvant therapy. The diagnostic criteria for differentiating MPLC from IPM relies on histological comparison of these multifocal tumors. However, the differentiation between them can be challenging when the multifocal tumors are histologically identical.^2^ Accumulating evidence suggest molecular profiling with next‐generation sequencing (NGS), employed to identify driver mutations that guide targeted therapy, has emerged as a helpful adjuvant in defining lineage in multiple lesions based on driver mutations such as *EGFR*, *KRAS*, and *BRAF*.^3^

Kirsten rat sarcoma viral oncogene homolog (*KRAS*), belonging to the family of rat sarcoma virus (*RAS*) genes, occurs in approximately 20%–25% of lung adenocarcinomas in Western Countries and in approximately 10%–15% of cases in Asia.^4^ We herein report the first case of a patient with two simultaneously resected metachronous adenocarcinomas in the right upper lobe, each with a distinct driver mutation in the *KRAS* gene.

### Case report

**Figure 1:**
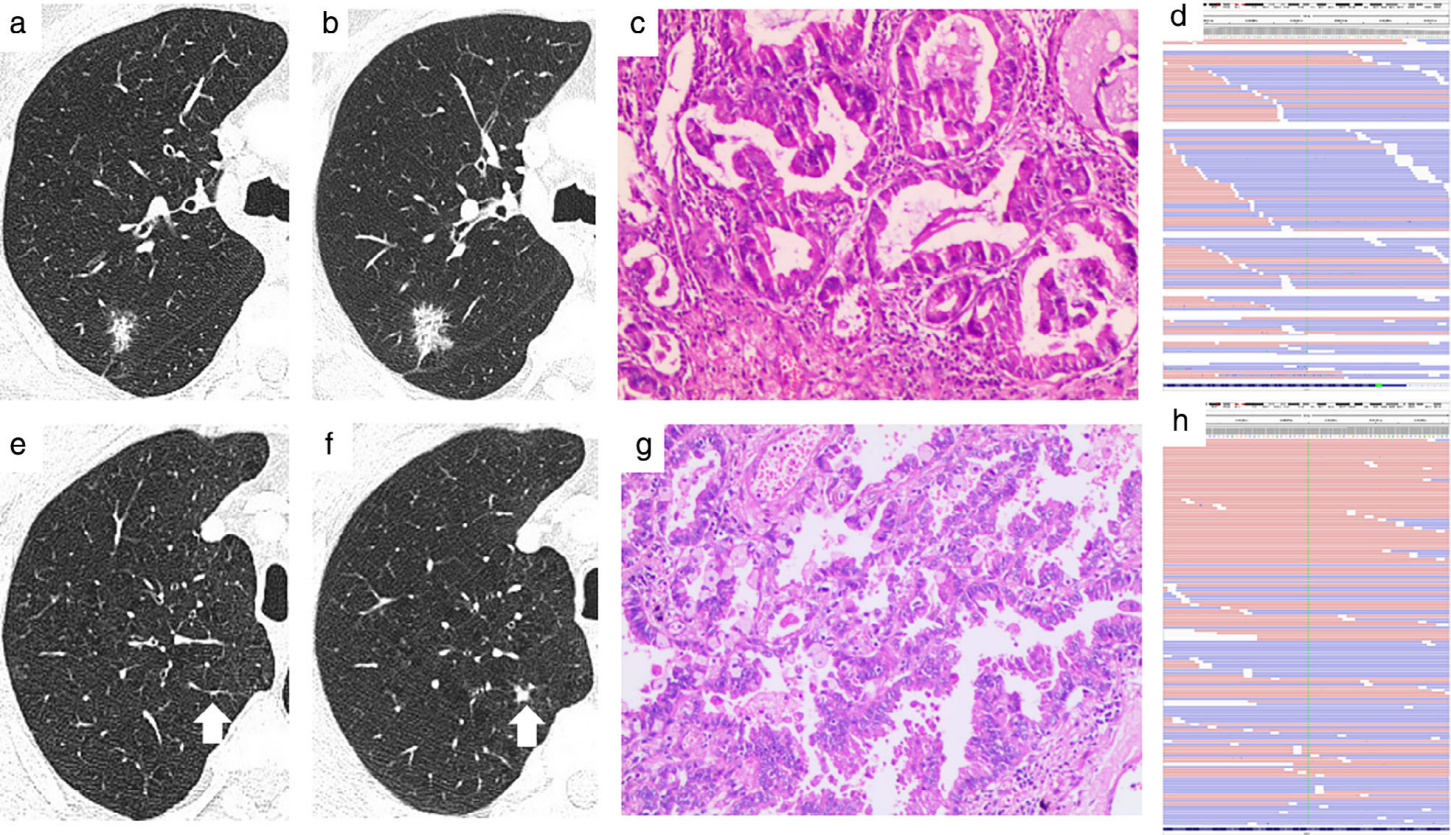
Radiographic, pathologic, and molecular findings of the metachronous nodules. Axial CT images showed chronological evolution of the metachronous lung cancers. (**a**) The nodule in the posterior segment of the right upper lobe (RS2) initially appeared as a small subsolid nodule of 1.8 cm maximum diameter without enhancement after contrast agent injection in June 2015, and over the next 44 months, and (**b**) enlarged to 2.3 cm with increased solid components and invasive features. The nodule in the apical segment of the right upper lobe (RS1) was not found in June 2015 (**e**), but emerged as a solid nodule of 0.8 cm maximum diameter in March 2019 (**f**). Both tumors revealed identical histology of moderately‐differentiated adenocarcinoma with lepidic growth (RS2 nodule [**c]**; RS1 nodule [**g**]). Molecular profiling showed distinct *KRAS* mutation in each tumor, with the RS2 tumor harboring the *KRAS*‐G12C mutation due to the c.34G > T nucleotide base substitution (**d**) and the other RS1 tumor harboring the *KRAS*‐Q61H mutation due to the c.183A > T nucleotide base substitution (**h**).

A 66‐year‐old male patient with a 30‐pack year smoking history was seen in our clinic with an incidental finding of a nodule in the right upper lobe (RUL) of the lung on routine health examination in June 2015. A chest CT scan was performed which indicated a small subsolid nodule of 1.8 cm maximum diameter in the posterior segment of the RUL (RS2), without enhancement after contrast agent injection (Fig 1a). No related subjective symptoms were noted and physical examination showed no abnormalities. Thus, little attention was paid to it by the patient apart from undergoing serial annual surveillance scans which showed no changes in the RS2 nodule until March 2019. A chest CT scan on March 2019 demonstrated the RS2 nodule had enlarged to 2.3 cm with increased solid components and invasive features (Fig 1b) as well as a newly‐present solid nodule of 0.8 cm maximum diameter in the apical segment of the RUL (RS1) (Fig 1f). The rapid progression following radiological presentation urged the patient to seek surgical treatment in our clinic, and who subsequently underwent video‐assisted RUL lobectomy and systematic lymphadenectomy. The postoperative course was uneventful. Histopathology demonstrated two nodules of moderately‐differentiated lepidic adenocarcinoma, without lymph node metastasis (Fig 1c and g), and no lymphovascular or perineural invasion was noted. This case was presumed to be stage IIB (T3N0M0) taking into consideration RS1 nodule intrapulmonary metastasis. Molecular analysis with NGS was performed using an institutionally‐developed panel of 1021 genes.^5^, ^6^ Sequencing revealed distinct *KRAS* mutation in each tumor, without other identical comutations (Table 1). The RS2 tumor harbored the *KRAS*‐G12C mutation due to the c.34G > T nucleotide base substitution (Fig 1d), while the other RS1 tumor harbored the *KRAS*‐Q61H mutation due to the c.183A > T nucleotide base substitution (Fig 1h). In addition, co‐occurring mutations in *STK11* and *TP53* were identified in the RS2 and RS1 tumors, respectively. Therefore, based on the distinct chronological evolution and molecular characteristics of both tumors, the patient was determined to have separate stage I primary lung adenocarcinoma and CT scan for monitoring was recommended, with no need for adjuvant chemotherapy. At six months postoperative follow‐up visit, the patient did not complain of any discomfort and CT scans of the chest and abdomen showed no abnormal signs.

**Table 1:** Mutational profile of the metachronous primary lung cancers

Nodule	Gene	Nucleotide changes	Functional region	Amino acid changes	Origin	Mutational type	Significance	VAF
RS2 nodule	IL7R	c.1173G > T	Exon 8	p.R391S	Somatic	Missense	Unknown	25.30%
	STK11	c.109C > T	Exon 1	p.Q37*	Somatic	Nonsense	Pathogenic	17.60%
	JAK2	c.1547G > A	Exon 12	p.G516D	Somatic	Missense	Unknown	15.70%
	EPAS1	c.1810A > G	Exon 12	p.M604V	Somatic	Missense	Unknown	15.50%
	KRAS	c.34G > T	Exon 2	p.G12C	Somatic	Missense	Pathogenic	14.40%
	IRF2	c.217G > C	Exon 4	p.D73H	Somatic	Missense	Unknown	13%
	NFE2L2	c.246A > C	Exon 2	p.E82D	Somatic	Missense	Unknown	2.30%
	FLT3	c.1773C > G	Exon 14	p.Y591*	Somatic	Nonsense	Unknown	2.30%
	POT1	c.186T > A	Exon 7	p.F62L	Somatic	Missense	Unknown	2%
RS1 nodule	TP53	c.725G > C	Exon 7	p.C242S	Somatic	Missense	Pathogenic	12.40%
	LRP1B	c.7457G > T	Exon 45	p.R2486I	Somatic	Missense	Unknown	11.90%
	TUBA3C	c.518C > A	Exon 4	p.P173Q	Somatic	Missense	Unknown	11%
	KRAS	c.183A > T	Exon 3	p.Q61H	Somatic	Missense	Pathogenic	9.90%
	CSF1R	c.2446C > T	Exon 19	p.R816C	Somatic	Missense	Unknown	9%
	LRP1B	c.13757T[2 > 1]	Exon 91	p.P4587Qfs*4	Somatic	Frame shift	Unknown	8.70%
	CHD8	c.589G > T	Exon 4	p.G197C	Somatic	Missense	Unknown	2.70%
	FLCN	c.812A > T	Exon 8	p.E271V	Somatic	Missense	Unknown	1.60%
	CCND1		All exon		Somatic	Amplification	Pathogenic	3.2

VAF, variant allele frequency.

## Discussion

The original criteria of distinguishing SPLC from IPM have been proposed by Martini and Melamed in 1975, based only on the tumor characteristics. Aside from the basic requirements of being temporally and physically distinct, metachronous multiple tumors must originate from the known carcinoma in situ, be located in different lobes or lung, or there should be a two‐year interval at least between the tumors if histologically identical, or they should be histologically different, to be considered metachronous MPLC.^7^ Some authors have modified this to at least a four‐year interval between histologically identical cancers and an interval of two to four years represents a gray area where it is difficult to decide whether a new lesion is a second primary.^8^ Therefore, decisions based on clinical and pathological data lack the necessary resolution to classify certain multiple lung cancers into either category, and may be subjective and affected by interobserver variability among chest physicians and pathologists.^9^ The International Association for the Study of Lung Cancer (IASLC) Lung Cancer Staging Project has recently proposed clinicopathological criteria to improve this distinction to lead to more consistent classification and clarity. However, they acknowledge that there is a substantial rate of misclassification using these criteria and a need for a multidisciplinary approach to consider all available information in the final staging.^10^

Molecular profiling has been explored as a means of refining multiple lung cancer staging. Cumulative evidence support the notion that molecular analysis of somatic changes within tumor DNA has emerged as a supplement in increasing the reliability of defining lineage of MPLC. Previous studies have reported as many as 32% of all histologically‐confirmed synchronous lung tumors have been misclassified as IPM compared with molecular analysis.^9^ Patients with lung adenocarcinomas are now routinely tested for a panel of oncogenic driver mutations (such as *KRAS*, *EGFR*, and *ALK*) in the clinical setting. These mutations are identified not only for the selection of precision therapy, but are of significant interest in the study of metachronous/ synchronous lung adenocarcinomas, as they are involved in early‐stage tumorigenesis before clonal expansion.^11^ Therefore, they should differ between primary lesions and metastases.

The clonal origin of multiple primary lung cancers is still a debated topic. Some authors have proposed a field cancerization theory, believing they originate independently from distinct progenitor cells.^12^ However, no definitive evidence has been reported to exclude the concept that MPLC arises from a single clonal event‐caused tumor that later spreads.^13^ Moreover, *KRAS* or *EGFR* mutations with identical or distinct mutant loci were shared in multifocal lesions in a study by Liu *et al*. which suggested that MPLCs have possible selection constraints around certain genes or pathways for tumorigenesis in specific patients.^14^ Another study further revealed functional interchangeability of different driver mutations in MPLC and also proposed simultaneous evolutionary expansion and constraint for functional convergence of tumorigenic pathways.^15^

*KRAS* is one of the most frequently mutated genes in NSCLC. RAS oncoproteins activate downstream cytosolic effectors that lead to uncontrolled cell proliferation and abnormal cell survival.^4^ The majority of *KRAS* mutations occur in codons 12 and 13, and *KRAS*‐G12C is the most frequent codon variant.^16^ Typically, *KRAS* mutations are found in tumors from heavy smoker patients.^16^ Mutations in tumor suppressor genes *TP53* and *STK11*, often co‐occurring with *KRAS* mutations, can predict a worse survival in the *KRAS*‐mutated lung adenocarcinoma when compared with *KRAS* mutation as a single aberration.^17^ Mutations in TP53 and *STK11* may contribute to the heterogeneity in *KRAS*‐mutant tumor biology, with *TP53* associated with enhanced proliferation and *STK11* with immune surveillance suppression.^18^ The case in our report initially presented a diagnostic and therapeutic dilemma as the tumors were in the same lobe, histologically identical, and detected between an interval of two to four years. Based only on the clinicopathological criteria, it is hard to define staging and determine whether adjuvant therapy should be initiated postoperatively. However, NGS provided us with additional molecular information on these tumors and allowed us to make correct lineage calling, thus correctly identifying which patients may benefit from adjuvant therapy.

## Disclosure

The authors have no conflicts of interest to declare.
